# Influences of smoking and caffeine consumption on trigeminal pain processing

**DOI:** 10.1186/1129-2377-15-39

**Published:** 2014-06-13

**Authors:** Dagny Holle, Anke Heber, Steffen Naegel, Hans-Christoph Diener, Zaza Katsarava, Mark Obermann

**Affiliations:** 1 Department of Neurology, University of Duisburg-Essen, Hufeland street 55, Essen 45147, Germany; 2 Department of Neurology, Ev, Hospital Unna, Unna, Germany

**Keywords:** Central pain processing, Inhibition, Facilitation, Nicotine, Caffeine

## Abstract

**Background:**

Many human and animal studies have shown the influence of nicotine and caffeine on pain perception and processing. This study aims to investigate whether smoking or caffeine consumption influences trigeminal pain processing.

**Methods:**

Sixty healthy subjects were investigated using simultaneous recordings of the nociceptive blink reflex (nBR) and pain related evoked potentials (PREP) following nociceptive electrical stimulation on both sides of the forehead (V1). Thirty subjects were investigated before and after smoking a cigarette, as well as before and after taking a tablet of 400 mg caffeine.

**Results:**

After smoking PREP showed decreased N2 and P2 latencies indicating central facilitation at supraspinal (thalamic or cortical) level. PREP amplitudes were not changed. NBR showed a decreased area under the curve (AUC) indicating central inhibition at brainstem level. After caffeine intake no significant changes were observed comparing nBR and PREP results before consumption.

**Conclusions:**

Smoking influences trigeminal pain processing on supraspinal and brainstem level. In the investigated setting, caffeine consumption does not significantly alter trigeminal pain processing. This observation might help in the further understanding of the pathophysiology of pain disorders that are associated with excessive smoking habits such as cluster headache. Previous smoking has to be taken into account when performing electrophysiological studies to avoid bias of study results.

## Background

The influence of caffeine and nicotine on pain processing is well described in the literature. Various analgesic as well as nociceptive properties of both substances were reported, previously. Smoking and caffeine consumption is quite common in the general population, and even more pronounced in some patient populations (e.g. smoking in cluster headache [1]. Caffeine itself can induce or exacerbate some pain entities (e.g. migraine [2], caffeine-withdrawal headache [3]). Furthermore, smokers are more prone to develop back pain, and general chronic pain conditions [4-14]. Additionally, higher pain intensity scores were reported in smokers [15].

To which extent smoking and caffeine consumption may influence pain specific trigeminal pain processing specifically remains unknown. Therefore, this study aims to investigate the caffeine and nicotine related alterations in trigeminal pain processing and its influence on electrophysiological study results. To assess the trigeminal nociceptive system, we used the nociceptive blink reflex (nBR) and trigeminal pain-related evoked potentials (PREP). These non-invasive electrophysiological study techniques allow nociception specific stimulation of the trigeminal pain processing system [16] and are highly sensitive for the detection of changes of trigeminal processing [17] at brainstem (nBR) as well as supraspinal (thalamic, cortical) level (PREP).

## Methods

### Standard protocol approvals, registrations, and patients consents

The study protocol was reviewed and approved by the Medical Ethics Committee of the University of Duisburg-Essen and written informed consent according to the Declaration of Helsinki was obtained from all participants prior to investigation.

### Subjects

Sixty healthy subjects were investigated in a longitudinal design. Thirty subjects were investigated before and 5 minutes after smoking a cigarette (mean age 27.2 ± 2.1 y; range 24–32), 30 subjects were investigated before and 15 minutes after taking 400 mg caffeine orally (mean age 27.9 ± 3.3 y; range 24–39). The amount of 400 mg caffeine equals approximately three cups of coffee. The subjects were instructed not to smoke, consume caffeine-containing beverages or eat within 4 hours prior to study participation.

Additional information on smoking habits and regular coffee consumption were inquired. Eighteen subjects in the smoking group smoked on a daily basis, 11 were social smokers (<3 days with smoking a week), one subjects was non-smoker. The mean caffeine consumption in the caffeine group was 157 mg ± 105 per day (range 0–400). Three groups of caffeine consumption were defined: 1.) no or irrelevant caffeine consumption (<30 mg/d; 3 subjects); 2.) moderate caffeine consumption (30–199 mg/d; 17 subjects); 3.) high caffeine consumption (>200 mg/d; 10 subjects).

### Electrophysiological settings

Two planar concentric electrodes (Walter Graphtek GmbH, Lübeck, Germany http://www.walter-graphtek.com/) were attached to the skin 10 mm above the entry zone of the supraorbital nerve. The outer rim of the first electrode was placed 1 cm from the forehead midline, the second electrode approximately 2 cm apart and lateral. Left side and right side were stimulated 15 times per session in each subject before and after smoking/caffeine consumption (triple pulse, monopolar square wave, duration 0.5 ms, pulse interval 5 ms, interstimulus interval: 12 to 18 seconds, pseudo-randomized). Starting side of stimulation was changed randomly. Perception and pain thresholds were determined on the forehead with an ascending and descending sequence of 0.2 mA intensity steps. The stimulus intensity was set at doubled individual pain threshold. Initial stimulus intensity was retained in the repletion after smoking or caffeine consumption. Stimuli were delivered to each side in pseudo random order in terms of start site (i.e., left or right side of the forehead).

NBR and PREP were recorded simultaneously following trigeminal stimulation of the forehead. The nBR was recorded using surface electrodes placed infraorbitally referenced to the orbital rim. Recording parameters: bandwidth 1 Hz to 1 kHz, sampling rate 2.5 kHz, sweep length 300 ms (1401 plus, Signal, Cambridge Electronic Design, UK). PREPs were recorded with electrodes placed at C_z_ referenced to linked earlobes (A1-A2) according to the international 10–20 system.

Signal analysis was performed by an investigator blinded to the intervention (caffeine/smoking). The first sweep was rejected to avoid contamination by startle response. The remaining 14 sweeps were averaged. For nBR onset latencies waveforms were rectified and analyzed for each sweep separately. A mean value for each session was calculated. Areas under the curve were calculated between 27 and 87 ms. Concerning PREP N2 (negative peak), P2 (positive peak) latencies and PPA (peak-to-peak) amplitudes were analyzed.

Mean values of results after right and left side stimulation and subsequent values for the group were calculated. Offline-analysis was performed with a custom-written PC-based software using Matlab (Matlab 7, The MathWorks, Natick, MA, USA).

### Statistical analysis

As of the longitudinal design paired t-test was used to compare mean values of AUC, nBR latency, PPA, N2 latency, and P2 latency before and after smoking as well as before and after caffeine consumption. Correlation between smoking habits as well as caffeine intake was determined. All statistics were calculated with SPSS 16 (SPSS Inc., Chicago, IL, USA). The level of significance was set to p < 0.05.

## Results

### Smoking

• *Nociceptive blink reflex:*

**Figure 1:**
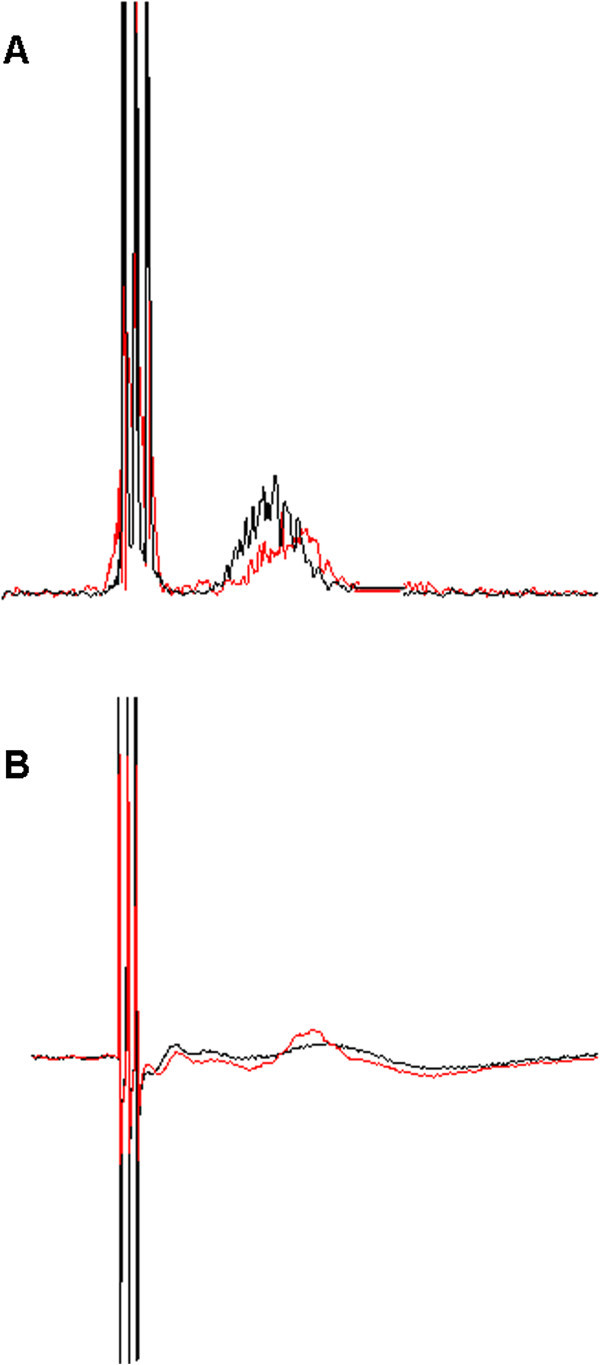
**Representative nBR and PREP curves before an after smoking in a single subjects*.*** NBR and PREP before smoking is shown in black. NBR and PREP after smoking is shown in red. **(A)** Area under the curve is decreased after smoking a cigarette suggesting an inhibition of trigeminal pain processing at brainstem level. **(B)** N2 and P2 latency are reduced after smoking a cigarette suggesting a facilitation of trigeminal pain processing at supraspinal level.

In all subjects the R2 response could be identified. Electrophysiological results of means are summarized in Table 1. Representative nBR responses are shown in Figure 1A. AUC was significantly decreased after smoking (Z df 29; p = 0.012), suggesting inhibition at brainstem level. Latency was unchanged before and after smoking. No correlation between smoking habits and electrophysiological results could be determined.

**Table 1:** Results of the nociceptive blink reflex before and after consuming caffeine 400 mg or smoking one cigarette

**Groups**	**Latency (ms)**	**Latency (ms)**	**Area under the curve (x10**^**3**^**) (ìV xms)**	**Area under the curve (x10**^**3**^**) (ìV xms)**
	***Before***	***After***	***Before***	***After***
Coffeine	47.49 ± 6.15	47.64 ± 6.33	119.82 ± 180.61	113.96 ± 183.24
	[36.65-63.40]	[35.70-71.75]	[24.18 ± 857.21]	[194.00-848.03]
Nicotine	46.00 ± 5.61	45.67 ± 4.39	110.52 ± 161.48	97.09 ± 140.1*
	[37.40-61.80]	[35.20-54.50]	[17.24-743.72]	[14.4-671.0]

*p < 0.05 t-test paired samples

After smoking Area under the curve is significantly reduced in terms of an inhibition of trigeminal pain processing at brainstem level. Caffeine consumption does not influence results of the nociceptive blink reflex. Results are presented as mean ± standard deviations and range.

• *Pain related evoked potentials:*

In 28 subjects PREP could be identified. Electrophysiological results of means are summarized in Table 2. Representative PREP responses are shown in Figure 1B. N2 latency (p = 0.002) and P2 latency (p = 0.022) were significantly reduced after smoking suggesting a facilitation at supraspinal level. Amplitudes were not significantly changed. No correlation between smoking habits and electrophysiological results could be determined.

**Table 2:** Results of the pain-related evoked potentials before and after consuming caffeine 400 mg or smoking a cigarette

**Groups**	**N2**	**N2**	**P2**	**P2**	***PPA***	**PPA**
	***Before***	***After***	***Before***	***After***	***Before***	***After***
Coffeine	182.60 ± 12.49	182.43 ± 11.24	131.78 ± 10.39	131.78 ± 9.60	52.03 ± 20.46	48.48 ± 22.64
	[165.90-211.70]	[161.35-206.70]	[108.90-52.80]	[115.00-153.00]	[19.70-128.30]	[16.45-144.70
Nicotine	182.15 ± 12.49	176.89 ± 10.36***	131.35 ± 9.35	128.32 ± 7.93*	51.55 ± 26.32	46.07 ± 22.63
	[158.30-205.40]	[161.30-199.10]	[155.75-150.40]	[113.00-141.70]	[10.40-117.30]	[13.70-84.45]

Results are presented as mean ± standard deviations and range.

*p < 0.05 t-test paired samples.

**p < 0.005 t-test paired samples.

After smoking N2 and P2 latency are significantly reduced showing a facilitation of trigeminal pain processing at supraspinal level.

### Caffeine consumption

• *Nociceptive blink reflex:*

In 28 subjects the R2 response could be identified. Electrophysiological results of means are summarized in Table 1. No significant changes regarding nBR latency and AUC were observed after caffeine consumption. No correlation between caffeine consumption and electrophysiological results could be determined.

• *Pain related evoked potentials:*

In all subjects PREP could be identified. Electrophysiological results of means are summarized in Table 2. No significant changes regarding N2 latency, P2 latency, and PPA were observed after caffeine consumption. No correlation of caffeine consumption and electrophysiological results could be determined.

## Discussion

Our results show inhibition at brainstem level and facilitation at supraspinal level of trigeminal pain processing after smoking a cigarette in healthy volunteers. After caffeine consumption no significant alterations were observed.

The effect of nicotine on event related potentials was investigated in several studies. In human pain models the effects of nicotine are often inconsistent and sometimes even conflicting. In cold pressor tests pain thresholds were increased by nicotine, whereas studies using heat or electrical stimulation pain models reported inconsistent or conflicting results [18]. Most of these studies showed a nicotine driven facilitation of brain processing in line with our findings [19-22]. In contrast to our results, Miyazaki et al. showed nicotine-related decreased amplitudes of laser evoked potentials [23]. The authors concluded that these alterations might reflect nicotine depended antinociceptive effects. However, a correlation of electrophysiological inhibition and subjective pain perception in the investigated subjects could not be established. One explanation for the variant study results might be the different stimulation methods, which are electric (i.e., direct skin nerve fiber depolarisation) and laser stimulation (i.e., indirect depolarisation of nerve fibers by skin nociceptors).

Another reason for this inconsistency of the effect of nicotine in humans might results from the fact that most studies investigate smokers, who probably display various kinds of habituation to the nicotinergic effects. Positron emission tomography (PET) studies showed an increase of AChRs (acetylcholine receptors) density compared to non-smokers and ex-smokers [24]. Chronic exposure induces widespread adaptive changes within the endogenous opioid system, which leads to measurable effects that can be subjectively reported by patients. For example, after tooth extraction, smokers needed more analgesics than non-smokers or light-smokers (<10 cigarettes a day) [25]. Pain sensitivity can be reduced by nicotine nasal spray or transdermal patch. Additionally, the rapid wash-in and wash-out period of nicotine after smoking as well as intake route (e.g. cigarettes, gum, intravenous, subcutaneous) and amount of nicotine absorption might make it difficult to investigate subjects during the exact same point of nicotine influence.

The observed alteration of trigeminal pain processing at brain stem level might be based on inhibition of trigeminal subnucleus caudalis neuronal responses by nicotine. Animal experiments showed an initial increase followed by a decline of firing rate of the trigeminal subnucleus that even persisted after reapplication of nicotine suggesting a pattern of self-desensitization [26].

The complexity and inconsistency of nicotine effects seems to offer an explanation for the observed parallel inhibiton at brain stem level and the facilitation at supraspinal level.

Unfortunately, our study can not differentiate whether acute or chronic nicotine exposure might have different impact on pain processing. It seems reasonable that long term smokers may react different when smoking a cigarette as further studies have to be performed to illuminate this interesting question. Additionally, more studies are also needed to analyse whether and how pain patients, for example cluster headache patients, show a different smoking dependent trigeminal pain processing compared with healthy controls.

Central effects of caffeine are well known and reported in many studies. Its influence on pain processing is well established. Caffeine is an antagonist of adenosine A_1_, A_2A_ and A_2B_ receptors [27], which are localized at multiple sites such as the spinal cord, the thalamus and other supraspinal sites [28]. Caffeine leads to augmentation of analgesic effects of several drugs such as acetaminophen [29,30], and several non-steroidal anti-inflammatory drugs (NSAIDs) Caffeine containing mixed analgesics are widely used in several pain disorders. However, in small doses caffeine-related hyperalgesia [31] and inhibition of analgesic effects of pain killers (e.g. acetaminophen [31,32], amitriptyline [33-36], carbamazepine/oxcarbazepine [37,38], and venlafaxine [35]) were reported. The different properties were explained by distinct receptor interaction. Although we did not show any influence of caffeine on trigeminal pain processing we cannot exclude any influence. Different dosages as well as other application regimes might lead to a divergent result. This might especially account for frequent intake.

Some limitations of our study have to be addressed. We did not measure nicotine or caffeine levels in the blood and, therefore, we cannot correlate blood levels with electrophysiological results. However, we chose measuring times and dosages according to previously conducted studies [23,39] and instructed patients to fasten 4 h before study participation. However, a time interval of 15 minutes after intake of caffeine might be too short to detect the entire effect of this substance. Additionally, we cannot exclude that different substances of content others than nicotine might also influence our study results. Furthermore, smoking is a more complex activity that not only includes nicotine intake. Other effects such as the tactile impression itself or influences on relaxation and reward systems might also be involved in its mode of action that might effect electrophysiological results.

One other major limitation is that we did not apply any placebo intervention. However, as we recorded a pattern of central facilitation and habituation only after smoking a sole habituation effect based on re-testing seems to be rather unlikely. Especially regarding smoking a placebo intervention would be quite difficult to establish. Our study design tries to reflect the reality of patients participating in electrophysiological studies as lifelike as possible.

## Conclusions

In summary, smoking displays direct impact on trigeminal pain processing which can be detected by electrophysiological investigations via nociceptive blink reflex and pain related evoked potentials. The exact mechanisms of this influence remain unknown, but might be involved in the pathophysiology of pain disorders such as cluster headache. Smoking should be considered a confounding factor in future electrophysiological studies. The period of time study participants should abstain from smoking and the influence of nicotine habituation in heavy and long-term smokers have to be investigated in the future.

## Abbreviations

nBR: Nociceptive blink reflex; PREP: Pain-related evoked potentials; PPA: Peak-to peak; nAChRs: Nicotine acetylcholine receptors.

## Competing interests

Dagny Holle has received a research grant from Grünenthal. Anke Heber has nothing to disclose. Steffen Nägel has nothing to disclose. Hans-Christoph Diener has received honoraria for participation in clinical trials, contribution to advisory boards or lectures from Addex Pharma, Allergan, Almirall, AstraZeneca, Bayer Vital, Berlin Chemie, Coherex Medical, CoLucid, Böhringer Ingelheim, Bristol-Myers Squibb, GlaxoSmithKline, Grünenthal, Janssen-Cilag, Lilly, La Roche, 3 M Medica, Minster, MSD, Novartis, Johnson & Johnson, Pierre Fabre, Pfizer, Schaper and Brümmer, SanofiAventis, and Weber & Weber; received research support from Allergan, Almirall, AstraZeneca, Bayer, Galaxo-Smith-Kline, Janssen-Cilag, and Pfizer. Headache research at the Department of Neurology in Essen is supported by the German Research Council (DFG), the German Ministry of Education and Research (BMBF), and the European Union. Zaza Katsarava has received research grants and honoraria from Allergan, Bayer, Biogen and Merck, and is an advisory board member for Allergan. Headache research at the Department of Neurology in Essen is supported by the German Research Council (DFG), the German Ministry of Education and Research (BMBF), and the European Union. Mark Obermann has received scientific grants by the German Federal Ministry of Education and Research BMBF 01EM 0513.

## Authors’ contributions

DH has planned and conducted the study, statistical analysis and interpretation of data. AH has conducted the electrophysiological investigations. SN has planned the study and helped with interpretation of data. HCD has planned the study and helped with interpretation of data. ZK has planned the study and helped with interpretation of data. MO has planned the study and helped with interpretation of data. After smoking N2 and P2 latency are significantly reduced showing a facilitation of trigeminal pain processing at supraspinal level. All authors read and approved the final manuscript
